# Codifying Collegiality: Recent Developments in Data Sharing Policy in the Life Sciences

**DOI:** 10.1371/journal.pone.0108451

**Published:** 2014-09-26

**Authors:** Genevieve Pham-Kanter, Darren E. Zinner, Eric G. Campbell

**Affiliations:** 1 Department of Health Management and Policy, School of Public Health, Drexel University, Philadelphia, Pennsylvania, United States of America; 2 Edmond J. Safra Center for Ethics, Harvard University, Boston, Massachusetts, United States of America; 3 Heller School for Social Policy and Management, Brandeis University, Waltham, Massachusetts, United States of America; 4 Mongan Institute for Health Policy, Massachusetts General Hospital, Boston, Massachusetts, United States of America; 5 Harvard Medical School, Boston, Massachusetts, United States of America; UCLA, United States of America

## Abstract

Over the last decade, there have been significant changes in data sharing policies and in the data sharing environment faced by life science researchers. Using data from a 2013 survey of over 1600 life science researchers, we analyze the effects of sharing policies of funding agencies and journals. We also examine the effects of new sharing infrastructure and tools (i.e., third party repositories and online supplements). We find that recently enacted data sharing policies and new sharing infrastructure and tools have had a sizable effect on encouraging data sharing. In particular, third party repositories and online supplements as well as data sharing requirements of funding agencies, particularly the NIH and the National Human Genome Research Institute, were perceived by scientists to have had a large effect on facilitating data sharing. In addition, we found a high degree of compliance with these new policies, although noncompliance resulted in few formal or informal sanctions. Despite the overall effectiveness of data sharing policies, some significant gaps remain: about one third of grant reviewers placed no weight on data sharing plans in their reviews, and a similar percentage ignored the requirements of material transfer agreements. These patterns suggest that although most of these new policies have been effective, there is still room for policy improvement.

## Introduction

In the life sciences, collegial sharing of research resources–data, methods, and materials–is believed to play a critical role in scientific progress [1]. Prompt and widespread dissemination of new methods and findings allows scientists to build on each other’s work quickly and to speed the advancement of science; failure to share can lead to needlessly duplicative research, unproductive lines of inquiry, and delays in scientific innovation.

Inherent in the practice of scientific sharing, however, are tensions between what is in the public interest and what is in the private interest of individual scientists [2], [3]. While science advances through the open dissemination of information, scientists are rewarded for their individual scientific contributions and ability to amass scientific priority and individual credit. Although there may be large public returns from scientists sharing with each other, there can be a large private cost to individuals from sharing in terms of lost scientific productivity and scientific lead, and lost opportunities for financial gain. These individual interests act as a brake or constraint on unfettered data sharing in the sciences.

Previous empirical research has shown that, despite the stated consensus of data sharing as a professional ideal, a great deal of secrecy and data withholding behavior still exists in the life sciences. In a 2000 survey, Campbell et al. showed that 44% of geneticists and 32% of other life scientists had engaged in some form of data withholding in the previous 3 years [4]. The desire to protect one’s scientific lead and preserve the proprietary value of one’s data were important factors in withholding behavior. Similar estimates–obtained through surveys, public data searches, and field experiments–have since been reported in various subfields of genetics and other life sciences [5]–[12].

Since Campbell’s original survey, there have been important data sharing policy developments within the life sciences. In an effort to encourage more data sharing, the NIH began requiring a data sharing plan in 2003 for grant applications with anticipated annual costs greater than $500,000 [13]. Other funding agencies and organizations, including the National Science Foundation (NSF), the Howard Hughes Medical Institute, and the Wellcome Trust, have followed suit [14]–[16]. In the area of genetics–beginning with the Human Genome Project where the importance of collaboration and the public goods effects of genomic data were abundantly clear early on–repositories such as the Database of Genotypes and Phenotypes, the Online Mendelian Inheritance in Man database, and the Database of Single Nucleotide Polymorphisms, were created to facilitate the sharing of data [17]. These types of repositories and other data sharing infrastructure and tools, such as online supplements, have also become more popular with journals as efficient ways of ensuring the dissemination of scientific information [18]. Most recently, the NIH issued a new Genomic Data Sharing (GDS) Policy, to be effective beginning 2015, that broadens genomic data sharing requirements for NIH-funded projects [19].

To examine the effects of these policy developments and changes in the data sharing environment that have emerged over the last decade, we fielded a survey of life science researchers in early 2013 on the topic of data sharing and withholding. This survey included many items that were identical to the 2000 Campbell data sharing and withholding survey [4] so we could assess changes in the practices and attitudes of researchers. We report our findings on trends of data sharing and withholding elsewhere [20]. In this paper, we focus on policies and new technologies that impinge on data sharing. In particular, we analyze the perceived effects of sharing policies of funding agencies and journals as well as of new sharing infrastructure and tools such as third party repositories and online supplements. We find that recently enacted data sharing policies and new data sharing infrastructure and tools have had a sizable effect on encouraging data sharing. In particular, third party repositories and online supplements as well as the data sharing requirements of funding agencies, particularly the NIH and the National Human Genome Research Institute (NHGRI), were perceived by scientists to have had a large effect on facilitating data sharing. In addition, we found a high degree of compliance with these new policies, although noncompliance resulted in few formal or informal sanctions. Despite the overall effectiveness of data sharing policies, some significant gaps remain: about one third of grant reviewers placed no weight on data sharing plans in their reviews, and a similar percentage ignored the requirements of material transfer agreements (MTAs). These patterns suggest that although most of these new policies have been effective, there is still room for policy improvement.

## Data and Methods

### Sample Selection

In 2013, we fielded a mail survey of academic life science researchers working in US institutions. We obtained a sample of 3000 researchers using a sampling strategy identical to that used by Campbell in 2000 [4].

Our sample consisted of researchers from four different strata: a clinical department stratum, a nonclinical department stratum, a genetics department stratum, and a Human Genome Project (HGP)/NHGRI investigator stratum. Three of the strata (clinical, nonclinical, and genetics) comprised researchers employed at US universities and medical schools identified as the top institutional recipients of extramural NIH support (more below). The fourth stratum consisted of individual recipients of HGP and/or NHGRI grants.

To derive the eligible population for the clinical, nonclinical, and genetics strata, we reviewed all NIH grants awarded during FY 2010 and identified the 100 US universities and medical schools that had been the top institutional recipients of these grants. We also identified the types of departments that received the most NIH funding, classifying them as either a clinical or a nonclinical department. We identified the top 5 clinical departmental types (internal medicine, psychiatry, pediatrics, pathology, and neurology) and the top 5 nonclinical departmental types (biochemistry, microbiology/immunology/virology, pharmacology, biology, and physiology). Because of our special interest in genetics, genetics departments were placed in a separate category in our classification system. We then randomly selected, at each of the top 100 institutions, one clinical department, one nonclinical department, and all genetics departments and programs (i.e., if two or more genetics programs existed within the same institution, all were selected into the sample).

After selecting the departments, we obtained the names, addresses, and telephone numbers of the primary research faculty in each department from departmental and university websites. We used this methodology to populate the clinical stratum, nonclinical stratum, and genetics stratum. Because of the survey’s focus on researchers, faculty members in the clinical stratum were eligible only if they had published at least one research article listed in the National Library of Medicine’s Medline database within the last 3 years.

We also added a fourth stratum consisting of principal investigators who had been directly funded by the HGP and/or the NHGRI in the last 5 years. These included researchers from the top 100 institutions previously identified (duplicates removed), other academic institutions, and independent research centers.

The final stratified sample of 3000 faculty members included all 483 investigators from the HGP/NHGRI stratum, 1317 faculty members in genetics departments (for a total of 1800 faculty members with some genetics association), 600 faculty members in selected nonclinical departments, and 600 faculty members in selected clinical departments. Faculty members in clinical, nonclinical, and genetics departments were selected at random from their respective strata.

### Survey Instrument Design and Administration

The design of the survey instrument was informed by 3 focus group discussions with geneticists and other life scientists, 10 semi-structured interviews with geneticists, a critical review by an expert panel of biomedical and social science researchers, and reviews of the literature. Focus groups comprising geneticists and other life scientists (e.g. immunologists, microbiologists, computational biologists) were convened at three large academic medical centers. The sampling frame for the focus groups consisted of all academic faculty working in genetics, human genetics, and other life science departments at these centers. Scientists were selected at random, with oversampling of geneticists, to receive invitations to participate in the focus groups. At each site, a focus group consisting of 6–8 scientists was formed from among those who agreed to participate.

The purpose of the focus groups was to provide updated definitions for the key concepts, variables, and questions to be used in the survey as well as to identify newly emerging themes in data sharing. A list of the seed questions asked during the focus groups is reported in Appendix S1.

Because some scientists may find it difficult to express their attitudes towards and experiences with data sharing and withholding with complete candor during focus group sessions, we also conducted 10 confidential, personal interviews. Scientists selected for one-on-one interviews were chosen from among individuals who had been invited to participate in focus groups but who had been unable to attend the focus group meeting. These semi-structured interviews broached topics and questions similar to those in the focus group meetings.

Using information gleaned from the focus groups, personal interviews, the original 2000 survey, and a review of the current literature, we developed the new instrument on data sharing and data withholding. This instrument included both questions from the 2000 survey and new survey items on topics that emerged during the focus groups, interviews, and literature review. An expert panel of biomedical and social science researchers was asked to critique the new survey instrument. After the changes suggested by the panel were incorporated into the survey, the instrument was pre-tested by Harris Interactive in September–October 2012 and fielded January–June 2013.

To maximize comparability with the previous survey and minimize mode effects, this survey was conducted in the same way as the 2000 survey, via mail. Subjects were sent a cover letter describing the study, the survey instrument, a postage-paid postcard, and a monetary incentive in the form of a check. They were asked to complete the survey anonymously and mail the completed survey and, separately, the postcard. Receipt of the postcard would allow Harris to confirm that the subject’s survey had been completed but would ensure respondents’ complete anonymity because the survey instrument had no unique identifying information. Nonresponse to the initial mail survey was followed up with a second mailing and up to 3 telephone calls. All elements of the survey protocol were approved by the Partners Human Research Committee.

### Response Rates

Of the 3000 life scientists in our original sample, 147 respondents were deemed ineligible because they had died, had retired, were on sabbatical, were out of the country, were not located at the sampled institution, or did not hold faculty appointments. Of the remaining 2853 eligible scientists, 1165 completed the survey, yielding a response rate of 41%. There was good representation in responses across the 4 strata sampled, with response rates of 35% (157/454) among NHGRI grant recipients, 42% (530/1262) among researchers in the genetics department sample, 38% (210/557) among researchers in the clinical department sample, and 46% (268/580) among researchers in the nonclinical department sample.

### Dependent Variables and Measures

The exact wording of the survey questions that were analyzed is reproduced in Appendix S2. We analyzed survey items related to the influence of funding agency policies, publication policies, intellectual property policies, informal policies, and training on respondents’ sharing of information and biomaterials with other academic scientists. Respondents were asked to rate whether these policies had a large influence against sharing, a small influence against sharing, no influence, a small influence towards sharing, or a large influence towards sharing.

We also analyzed items related to data sharing infrastructure and tools such as online supplements and third party repositories. In particular, we examined the frequency with which researchers submitted information using these tools (yes or no) and ratings of whether these infrastructure and tools hindered or helped respondents’ research (hindered a lot, hindered a little, no effect, helped a little, helped a lot).

Finally, we looked at questions related to respondents’ experience with the data sharing requirements and restrictions of genome-wide association studies and MTAs.

### Statistical Analyses

All proportions reported in the text, tables, and figures were weighted to account for different probabilities of selection from the four strata and for nonresponse. All analyses were done using Stata/SE 13.1 (StataCorp, College Station, TX).

## Results

Table 1 reports the characteristics of researchers for the full sample and for the 4 subsamples (NHGRI principal investigators, faculty in genetics departments, faculty in clinical departments, and faculty in nonclinical departments). In the full sample, 29% of respondents were female, and 80% had been trained in the US. Most respondents reported their highest degree to be a PhD (75%), with a substantial minority (14%) reporting an MD degree, and 9% reporting both MD and PhD degrees. There was a wide range of professional experience represented in the sample: 13% had received their highest degree fewer than 10 years ago while 29% had received their degrees more than 30 years ago. About half of the full sample were full professors.

**Table 1:** Characteristics of survey respondents.

	Number of Respondents (%)				
Variable	Full Sample	NHGRI Subsample	Genetics Subsample	Clinical Subsample	Nonclinical Subsample
**Gender**					
Female	342 (29%)	42 (27%)	162 (31%)	73 (35%)	65 (24%)
Male	809 (69%)	111 (71%)	362 (68%)	135 (64%)	201 (75%)
**Highest degree**					
MD	162 (14%)	14 (9%)	37 (7%)	97 (46%)	14 (5%)
PhD	873 (75%)	121 (77%)	436 (82%)	84 (40%)	232 (87%)
MD-PhD	101 (9%)	13 (8%)	40 (8%)	27 (13%)	21 (8%)
**Trained in the US**					
Yes	930 (80%)	133 (85%)	431 (81%)	161 (77%)	205 (76%)
No	226 (19%)	21 (13%)	96 (18%)	48 (23%)	61 (23%)
**Years since highest degree**					
0–5	32 (3%)	5 (3%)	11 (2%)	11 (5%)	5 (2%)
6–10	117 (10%)	15 (10%)	53 (10%)	33 (16%)	16 (6%)
11–20	354 (30%)	51 (32%)	151 (28%)	61 (29%)	91 (34%)
21–30	310 (27%)	45 (29%)	156 (29%)	54 (26%)	55 (21%)
31–40	231 (20%)	26 (17%)	108 (20%)	30 (14%)	67 (25%)
>40	101 (9%)	8 (5%)	42 (8%)	19 (9%)	32 (12%)
**Academic rank**					
Full professor	567 (49%)	90 (57%)	262 (49%)	75 (36%)	140 (52%)
Associate professor	299 (26%)	40 (25%)	136 (26%)	49 (23%)	74 (28%)
Assistant professor	237 (20%)	20 (13%)	113 (21%)	57 (27%)	47 (18%)
Instructor or Lecturer	33 (2%)	1 (0.6%)	7 (1%)	24 (11%)	1 (0.4%)
**Publications in last 3 years**					
0–5	337 (29%)	16 (10%)	174 (33%)	75 (36%)	72 (27%)
6–15	484 (42%)	62 (39%)	229 (43%)	75 (36%)	118 (44%)
>15	264 (23%)	63 (40%)	96 (18%)	47 (22%)	58 (22%)
**Human subjects research in last 3 years**					
Yes	446 (38%)	73 (47%)	167 (32%)	150 (71%)	56 (21%)
No	711 (61%)	82 (52%)	360 (68%)	60 (29%)	209 (78%)
***Sample size***	1,165	157	530	210	268

Note: Percentages may not sum to 100% because of item non-response.

Across the different subsamples, respondent characteristics were similar along most dimensions, with the exception of: the highest degree held, the number of publications in the last 3 years, and whether the respondent was involved in human subjects research. Not surprisingly, those in the clinical subsample were more likely to hold MD degrees and were more likely to be involved in human subjects research; respondents in the NHGRI subsample reported more publications than those in the other subsamples.

### Influence of Data Sharing Policies

**Figure 1:**
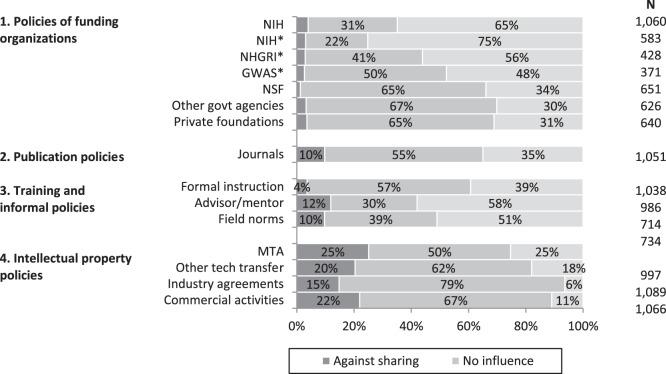
Influence of policies on data sharing.

Of the new data sharing policies that have been enacted since 2000, the policies of NIH were reported to have had the greatest impact on facilitating data sharing. As Figure 1 shows, 65% of respondents thought that NIH policies had been influential in increasing data sharing. NIH policies were rated particularly highly among self-identified geneticists (indicated by asterisks in the figure), with 75% of genetics researchers rating these policies as having been influential in facilitating data sharing. Among geneticists, NHGRI and Genome-Wide Association Study (GWAS) policies were thought to also facilitate data sharing, but to a lesser degree, than other NIH policies. Policies of non-NIH funding organizations such as NSF, other government agencies, and private foundations had a modest impact on increasing data sharing (31%–34%).

Journal publication policies were perceived by scientists to have had only a moderate effect on data sharing: 35% of respondents reported that journal publication policies had a positive influence towards facilitating data sharing. More than half (55%), however, reported that these policies had had no influence on sharing. Individual instruction through formal courses also had only a modest effect on data sharing, with 39% rating formal instruction as having had an influence on encouraging sharing. Informal training, however, through advisors or as reflected through the practices of others working in the field, were thought to have had a greater influence: 58% and 51% of respondents rated the practices of advisors and the norms of their field, respectively, as having been influential in encouraging data sharing.

In addition to policies that have been developed to encourage data sharing, there has also been the expansion of policies that discourage sharing. Intellectual property policies, which are put in place to protect the potential financial interests of universities and firms, impose conditions on what information and materials can be shared, thereby acting as a brake on scientific sharing. The bottom panel of Figure 1 shows respondents’ ratings of the influence of intellectual property policies such as MTAs, technology transfer agreements, and industry nondisclosure agreements. The larger shares of the bars shaded black show that intellectual property policies, relative to the policies discussed earlier, have had a substantial influence *against* data sharing. Twenty percent of respondents thought that institutional material transfer agreements had been influential in discouraging data sharing, and 25% of respondents thought the same of other technology transfer policies. Similarly, industry agreements and commercial activities (such as potential patents and royalties) were thought to impede sharing. These findings suggest that industry relationships have somewhat stronger negative effects on data sharing than found in recent research [6], [11].

### Compliance With Policy Requirements

Compliance with sharing requirements varied across domains and policies. We asked respondents about their compliance with the sharing policies of professional journals and, if applicable, GWAS policies and MTAs at their institutions. In general, there was a high degree of compliance with journals’ requirements related to the sharing of methods, data, and biomaterials. Almost all respondents (92%) reported always having submitted, when required to do so, a detailed description of their methods as an online supplement; 8% of respondents only sometimes submitted this description. Slightly lower percentages reported always submitting, when required, data as an online supplement (89%) or to a third party repository (90%). There was somewhat less compliance with submitting biomaterials to a third party repository (83%).

Researchers who conduct a GWAS funded by NIH are required to deposit data from the study into a designated repository, the Database of Genotypes and Phenotypes (dbGaP). dbGaP makes the information from these studies immediately available to the public; as an incentive for prompt data sharing, researchers are given a 12-month exclusivity period in which they and their collaborators are the sole authors permitted to publish work based on this data [21]. In this survey, we asked self-identified geneticists about data sharing related to GWAS. Among respondents who had conducted or collaborated on a GWAS in the last 3 years (n = 124), 75% reported being required to deposit data into a third party repository. Among those required to deposit data, 96% complied with this requirement. In addition, we found that most GWAS researchers were able to take advantage of the exclusivity period: 77% of those who submitted their data were able to submit their first publication using this data within 12 months.

Whereas journals and funding agencies have focused on policies that expand data sharing, universities and academic medical centers have concerned themselves with preserving institutional intellectual property, a policy objective that can conflict with open, unfettered data access. Institutionally-required MTAs and other technology transfer agreements have therefore been viewed among scientists as imposing restrictions on data sharing rather than facilitating sharing [22]. In our survey, we found substantial failure to comply with requirements related to MTAs. Table 2 shows the frequency of compliance to MTAs and the reasons for noncompliance. When respondents were asked how frequently they shared data or materials without an MTA even though they knew that such an agreement was required, 24% said that they sometimes or always violated MTA policies (57% said they never did). An additional 9% of respondents were not at all aware of the policies at their university related to MTAs. Much of MTA noncompliance can be attributed to the bureaucratic difficulties of obtaining an MTA rather than to philosophical objections. About 85% of respondents cited the time required to set up an MTA as a very important or moderately important reason for noncompliance, while 82% cited the red tape and 78% cited the onerousness of MTA negotiations. This compares to the relatively fewer researchers who attributed their noncompliance to a philosophical opposition to MTAs (48%) and the overly broad scope of MTAs (38%).

**Table 2:** Frequency and reasons for MTA policy violations.

A. Frequency of MTA policy violations	Weighted % (N = 993)
Always	5%
Sometimes	19%
Rarely	10%
Never	57%
Not aware of MTA policies	9%
	**Weighted % Indicating**
	**Very Important or**
**B. Reasons for violating policy** *	**Moderately Important Reason**
MTA takes too much time	85%
MTA requires too much red tape	82%
MTA negotiations too onerous	78%
Philosophically opposed to MTA restrictions	48%
Scope of MTA overly broad	38%

*among those who reported having violated institutional MTA policies.

### Policy Tools and Infrastructure Influencing Data Sharing

#### Data sharing plans in grant proposals

One of the biggest changes in the last decade has been the 2003 NIH policy that required all NIH grant applications with annual costs exceeding $500,000 to include data sharing plans [13]. Other federal agencies such as NSF have enacted similar policies [14]. This survey is, to our knowledge, the first to ask how proposal reviewers weight these data sharing plans in their evaluations. In the survey, we asked respondents whether they had served as a grant reviewer for federal agencies on life science research proposals and how important data sharing plans had been in their evaluation of proposals. Of the 735 respondents who had served as grant reviewers in the last 3 years, 27% said that the quality of the data sharing plans had been important or very important in their evaluation of proposals; an additional 43% said that the plans had been somewhat important. At the same time, a large minority (30%) said data sharing plans had not been at all important in their review of proposals, suggesting that some researchers may not be supportive of data sharing or of the use of NIH grants policy to facilitate data sharing.

#### Data sharing infrastructure and tools

Many data sharing policies that have been enacted in the last 10 years have been buttressed by the development of data infrastructure and tools such as online supplements and third party repositories. Active researchers may on occasion benefit from these supplements and repositories but may also find them burdensome since researchers must also contribute to them. We asked respondents to rate the degree to which supplements and repositories had helped or hindered their research. Because this global measure combines respondents’ experience as both users and contributors, it can be interpreted as the degree to which researchers are *net* beneficiaries of these supplements and repositories, that is, whether the benefits outweigh the cost of compliance.

**Figure 2:**
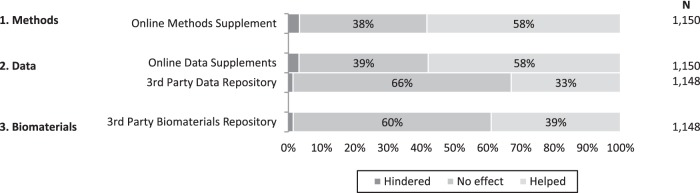
Effect of data sharing tools progress of research.

Figure 2 shows the degree to which researchers believed that these tools and new infrastructure had helped or hindered the progress of their research. In general, online data and methods supplements were thought to have helped respondents’ own research: 58% of respondents thought that these online supplements had been helpful. However, the effect of third party repositories was more muted. About one third of respondents thought that third party data repositories had helped their research progress, and 40% thought that third party biomaterials repositories had helped.

### Sanctions for Noncompliance

One intriguing finding is that there appear to be very few formal or informal sanctions for data sharing noncompliance; if a scientist fails to share as required or expected, she or he faces few penalties from other scientists. In our survey, when respondents were asked about whether they had appealed to a funding agency, journal, or professional association in response to another scientist’s failure to share data or biomaterials, only 4% said that they had. Instead of moving for formal sanctions against noncompliant colleagues, scientists more frequently imposed informal sanctions, although these informal sanctions were also infrequent. For example, when faced with an academic colleague’s failure to share information or materials, 17% of respondents said that they had stopped collaborating with the nonsharing colleague. Eight percent had taken steps to delay sharing, and 5% had refused to share their own data with the noncompliant scientist.

## Discussion

Over the last decade, life science researchers have faced significant changes in policies that govern data sharing and in the data sharing environment [20]: NIH codified the importance of data sharing by requiring data sharing plans of many of its grant applications; more journals have begun requiring publication of online supplements of data and methods; third party repositories for data and biomaterials have become available; and universities have begun requiring formal agreements before their researchers can share data with others outside of their home institution. In this paper, we report on how these policy changes have, from the perspective of life science researchers, influenced data sharing and data withholding practices.

Because this survey inquired about a wide range of policy developments, we were able to describe the scope and direction of influence of many different policy changes. Although a full characterization of the mechanisms underlying these effects is beyond the purview of the survey, we have found it helpful to organize our interpretations using the models of scientific organization and production provided by Dasgupta & David and Stephan [3], [23]. These primarily economic models, which also incorporate psychological and sociological theory, present a useful framework for thinking about scientists’ decisions to share–decisions based on private returns and relative costs and benefits. These models are also helpful for thinking about socially optimal infrastructure and conditions. Given the available data in our survey and in the existing literature, our discussion of mechanisms is necessarily speculative but can be a useful basis for organizing further empirical investigation.

Our survey and analysis point to three important effects of these policy developments. First, NIH policies have had a strong influence on increasing data sharing in the life sciences. These findings build on the work of Piwowar, who found that authors of studies that were funded by a large number of NIH grants were more likely to share their study raw data [8]. We conjecture that the NIH policy effect occurred through several different pathways. The two primary NIH policies enacted during this period were the requirement of a data sharing plan as part of the grant application process [13] and the NHGRI GWAS requirement of depositing GWAS findings in the dbGaP repository [21]. These NIH policies could have exerted their strong influence through:

- establishing a new default norm of treating new research data as a public resource to be shared rather than as a private asset owned by the originating research group;
- making researchers more conscious of and thoughtful about data sharing and requiring them to specify and take concrete actions to share new data;
- when projects were equally competitive, favoring projects with data sharing plans during grant review and penalizing projects with underdeveloped data sharing plans;
- spurring the use of dbGaP and other third party repositories.

In effect, NIH data sharing policies changed the relative returns of public and private incentives to sharing. By linking data sharing to positive things–such as favorable grant review and data release to third party repositories, which are a less costly form of sharing for the researcher in terms of time and effort–NIH lowered the private cost of sharing. In addition, since everyone was now expected to share, scientists who had previously shared while others did not no longer asymmetrically bore the cost of sharing. Put differently, by requiring data sharing of *all* NIH-supported scientists, NIH leveled the playing field so that withholding scientists would not benefit at the expense of sharing scientists.

By broadening data sharing requirements of NIH-supported scientists, the new NIH GDS policy reinforces this sharing norm. At the same time, however, the policy also abolishes the GWAS 12-month exclusivity/embargo period [19], thereby increasing the private cost of sharing and possibly delaying sharing. The net effect on this specific type of genomic data sharing will be important to monitor and report.

A second related point is that an important part of the effectiveness of funding agency policies and journal policies may lie in reducing the administrative and procedural burdens of sharing. Funding agencies approve data sharing plans that promise the release of data or biomaterials to a third party repository or the posting of data on public websites [13]–[16]. Journal policies require the publication of methods or data supplements online [5]. Third party repositories and online supplements reduce the cost of sharing by:

- reducing the administrative time and effort costs for the researcher providing the data because s/he only has to make the data available once, instead of fulfilling individual requests separately;
- reducing administrative costs for the providing researcher by having a third party screen and process requests;
- reducing administrative costs for the requesting researcher because s/he does not need to go through separate procedures to contact the originating scientist and will be vetted only once by a repository.

This standardization and reduction in costs for both data provider and requestor leads to greater participation in the data sharing process, as Tenopir et al. have highlighted [10]. In addition, because the process is more standardized, sharing may be more inclusive and fair, not dependent on the kinds of personal relationships and favoritism that may have characterized sharing through individual requests.

Finally, we have been able to identify some significant policy gaps and tensions. Our survey is, to our knowledge, the first to investigate quantitatively three aspects of data sharing tied to the emergence of new policies: (1) the importance that reviewers place on data sharing plans; (2) the degree to which sanctions are imposed for data sharing noncompliance; and (3) the degree of compliance with institutional MTA policies.

Our finding that almost one third of grant reviewers did not consider data sharing plans to be at all important is surprising. That such a high proportion of reviewers ignore data sharing plans suggests that many leading scientists do not view the dissemination of raw data or intermediate materials to be a responsibility accompanying the creation of new knowledge. Or, at the very least, they do not consider them to have the same importance as the dissemination of scientific findings or results. Perhaps these scientists support data sharing in principle but do not think data sharing plans tied to grant proposals are the means through which sharing should be encouraged; or they believe it is not their role as external reviewers to evaluate such plans and cede this role to NIH program staff; or they are not enthusiastic supporters of data sharing in general. This will be an important area to investigate further, particularly now that data sharing plans occupy a more central place, at least formally, in the new NIH GDS policy.

A second surprising finding is that there appear to be few sanctions or penalties for noncompliance. Scientists who interact with noncompliant scientists rarely go through formal appeals processes either to report the noncompliance or to ensure compliance. In addition, scientists infrequently sanction noncompliant scientists through informal means, such as breaking off collaborations. Both economic and sociological theory suggest that policies are most effective when there is enforcement of policies or the credible threat of sanctions [24], [25]. A reasonably high degree of compliance without apparent threat of sanctions is a puzzle that warrants further study. It could be the case that, because the policies are relatively new, there is initial compliance, but as scientists are learning what actions will or will not be sanctioned, the average level of compliance may change. It could also be the case that norms have developed around a high level of data sharing, but even so, sociological theory tells us that informal sanctions are often necessary to sustain norms [25]. Or there may be other informal sanctions being imposed that our survey did not detect; noncompliant scientists may be subject to, say, social isolation or harsher review in intangible ways during the publication or grants process. Finally, it may be that the sharing that is influenced by certain policies is not of a nature that is substantively helpful to scientists. For example, although there was a high degree of self-reported compliance with journal publication policies, only one third of scientists indicated that these policies had a positive influence on sharing. This discrepancy may reflect the possibility that scientists would have shared their data even without the policies, but it may also reflect perfunctory compliance: data that are made available are low-quality, disorganized, or poorly annotated and therefore difficult to interpret and re-use. In other words, a lack sanctioning for data sharing noncompliance may mean that what is being shared may not be all that useful. This scenario is consistent with previous work by Alsheikh-Ali et al [5].

A third tension is reflected in our finding that more than one third of scientists had either violated their institution’s MTA policy in the last 3 years or did not know the policy. This large-scale noncompliance is likely to be dismaying news for many universities, which had implemented these policies in the hope of providing a structure for balancing scientific needs for sharing with university intellectual property interests [22], [26]. The sheer scale of these violations suggests that current institutional MTA policies have not yet found the right balance, and may be too heavily weighted towards institutional interests at the expense of scientists’ professional and scholarly needs. We note that most researchers who knowingly violated their university’s MTA policies attributed their noncompliance to the procedural burden of MTAs. Given the apparent success of repositories in alleviating these kinds of burdens for researchers, the creation of a central clearinghouse/repository for MTA requests could improve compliance.

There are several limitations to this study. Our study may underestimate the degree of data withholding and compliance; even though respondents were informed that their survey responses would be completely anonymous, some social desirability bias may still exist if respondents underreport withholding and other behaviors that do not conform to scientific ideals. Second, researchers’ perceptions of the effects of policies may diverge from the actual effects of policies on researcher behaviors, although one could argue that perceptions of policy effects are important in and of themselves. Third, we did not ask respondents about their knowledge of data sharing policies, so some answers–for example, those about the effectiveness (or ineffectiveness) of policies–may be attributable to researchers not knowing policies rather than to their perception of policy consequences. Fourth, we did not evaluate the quality of data, methods, or materials that were posted online or submitted to third party repositories. It may be possible, as discussed earlier, that researchers made only cursory efforts to comply with data sharing rules and submitted low-quality or incomplete information. Fifth, there may be bias from non-response in that non-responders may be different from responders in systematic ways that relate to data sharing or withholding. Finally, our sample consisted of research universities; different patterns of sharing behavior and policy influences may be present at less research-intensive universities, so our findings may not extend to these types of institutions.

Overall, the public goods feature of data sharing points to an important role to be filled by actors like funding agencies and journals that have a public interest in moving science forward. These external actors can do what no single private party has an incentive to do or is able to do on its own: decrease the individual costs of sharing and level the playing field. Our analysis points to the NIH and, to a somewhat lesser degree, journals and other funding agencies, successfully able to play the role of an independent third party norm-setter and enforcer. Their policies and accompanying sharing tools have had substantial positive impact on the sharing and availability of data and biomaterials and on the progress of the research of individual scientists. There remains a need for policy refinements, however. That NIH policies have had a significant positive effect suggests that funding agencies in general can have important leverage on data sharing. The success of repositories in reducing costs and the increase in their use and compliance suggests that this kind of model is to be encouraged. Tensions in the role of data sharing in the grant review process, in enforcing policies, and in how to balance between institutional intellectual property rights and scientists’ norms and needs, will need to be clarified and resolved as science and scientific sharing models evolve.

## Supporting Information

Appendix S1 **Focus Group Seed Questions.**
(PDF)

Appendix S2 **Survey Questions Used in Analysis.**
(PDF)
