# Supplementary material for: Codifying Collegiality: Recent Developments in Data Sharing Policy in the Life Sciences
Source: PLoS One. 2014 Sep 26;9(9):e108451. doi: 10.1371/journal.pone.0108451 (PMC4178158; doi:10.1371/journal.pone.0108451)
Supplement: Appendix S1 — Focus Group Seed Questions. (PDF) [file pone.0108451.s001.pdf]

Codifying Collegiality: Recent Developments in Data Sharing Policy in the Life Sciences

Genevieve Pham-Kanter, Darren Zinner, and Eric G. Campbell

Supplementary Appendix S1. Focus Group Seed Questions

1. What are the various ways that scientists can withhold data in science today?

2. What types of data tend to be shared and which tend to be withheld?

3. How have data sharing and withholding practices changed in the last decade?

4. What factors affect the tendency of genetics researchers to share data?

5. How have those factors changed in the last decade?

6. How aware are researchers of government and university policies related to data sharing and data withholding?

7. How have data sharing and withholding among scientists changed in recent years?

8. What impact has data withholding had on your individual and your laboratory's productivity?

9. How aware are researchers of various federal policies related to data sharing and withholding and how often do they comply with these policies?
