# Supplementary material for: Codifying Collegiality: Recent Developments in Data Sharing Policy in the Life Sciences
Source: PLoS One. 2014 Sep 26;9(9):e108451. doi: 10.1371/journal.pone.0108451 (PMC4178158; doi:10.1371/journal.pone.0108451)
Supplement: Appendix S2 — Survey Questions Used in Analysis. (PDF) [file pone.0108451.s002.pdf]

Codifying Collegiality: Recent Developments in Data Sharing Policy in the Life Sciences

Genevieve Pham-Kanter, Darren Zinner, and Eric G. Campbell

Supplementary Appendix S2. Survey Questions Used in Analysis

INFLUENCE OF POLICIES OF FUNDING ORGANIZATIONS

1. Overall, how much influence does each of the following policies of funding organizations have on your sharing of information, data or materials with other academic scientists?

	Large influence *against* sharing	Small influence *against* sharing	No influence	Small influence *towards* sharing	Large influence *towards* sharing	Not applicable
a. Overall data sharing policies of the National Institutes of Health (NIH)	☐1	☐2	☐3	☐4	☐5	☐6
b. Data sharing policies of the National Human Genome Research Institute (NHGRI)	☐1	☐2	☐3	☐4	☐5	☐6
c. NIH policies governing genome-wide association studies (GWAS)	☐1	☐2	☐3	☐4	☐5	☐6
d. Data sharing policies of the National Science Foundation (NSF)	☐1	☐2	☐3	☐4	☐5	☐6
e. Data sharing policies of government agencies-other than NIH or NSF (e.g., Department of Defense, USDA)	☐1	☐2	☐3	☐4	☐5	☐6
f. Policies of private funding organizations and foundations (e.g., HHMI, Wellcome Trust)	☐1	☐2	☐3	☐4	☐5	☐6

INFLUENCE OF OTHER POLICIES (PUBLICATION POLICIES, TRAINING AND INFORMAL POLICIES, INTELLECTUAL PROPERTY POLICIES)

2. Overall, how much influence does each of the following have on your sharing of information, data or materials with other academic scientists?

	Large influence *against* sharing	Small influence *against* sharing	No influence	Small influence *towards* sharing	Large influence *towards* sharing	Not applicable
a. Attitudes and practices of former advisors or mentors	☐1	☐2	☐3	☐4	☐5	☐6
b. Formal instruction you received while in training (Responsible Conduct of Research training, classes, seminars, etc.)	☐1	☐2	☐3	☐4	☐5	☐6
c. Policies governing material transfer agreements (MTAs) at your institution	☐1	☐2	☐3	☐4	☐5	☐6

d. Technology transfer policies, other than MTAs, at your institution	$\square_1$	$\square_2$	$\square_3$	$\square_4$	$\square_5$	$\square_6$
e. Agreements with industrial sponsors	$\square_1$	$\square_2$	$\square_3$	$\square_4$	$\square_5$	$\square_6$
f. Your commercial activities (i.e., patenting, licensing, etc.)	$\square_1$	$\square_2$	$\square_3$	$\square_4$	$\square_5$	$\square_6$
g. Publication policies of professional journals	$\square_1$	$\square_2$	$\square_3$	$\square_4$	$\square_5$	$\square_6$
h. Informal rules/norms of your research field	$\square_1$	$\square_2$	$\square_3$	$\square_4$	$\square_5$	$\square_6$

COMPLIANCE WITH JOURNAL POLICIES

3a. Please indicate whether, in the past 3 years, you have been *required* **by a journal to…?** *For each "yes" response, please answer question <3b>.*

3b. How often did you submit, make available or transfer this information?

	No	Yes		Never	Sometimes	Always	Not applicable
a. Submit detailed description of methods as online supplement	$\square_1$	$\square_2$	→	$\square_1$	$\square_2$	$\square_3$	$\square_4$
b. Make data available as online supplement	$\square_1$	$\square_2$	→	$\square_1$	$\square_2$	$\square_3$	$\square_4$
c. Submit biomaterials (e.g., tissues, reagents, organisms, etc.) to third party repository	$\square_1$	$\square_2$	→	$\square_1$	$\square_2$	$\square_3$	$\square_4$
d. Submit data to third party repository (e.g., GenBank)	$\square_1$	$\square_2$	→	$\square_1$	$\square_2$	$\square_3$	$\square_4$
e. Transfer biomaterials (e.g., tissues, reagents, organisms, etc.) to accommodate request from another lab	$\square_1$	$\square_2$	→	$\square_1$	$\square_2$	$\square_3$	$\square_4$
f. Make data available to accommodate request from another lab	$\square_1$	$\square_2$	→	$\square_1$	$\square_2$	$\square_3$	$\square_4$

EFFECT OF JOURNAL REQUIREMENTS ON PROGRESS OF RESEARCH

4. Overall, how much has each of the following hindered or helped the progress of your research?

	Hindered [illegible]	Hindered [illegible]	No effect	Helped [illegible]	Helped [illegible]
a. Online methods supplements	$\square_1$	$\square_2$	$\square_3$	$\square_4$	$\square_5$
b. Online data supplements	$\square_1$	$\square_2$	$\square_3$	$\square_4$	$\square_5$
c. Third party biomaterials repositories	$\square_1$	$\square_2$	$\square_3$	$\square_4$	$\square_5$
d. Third party data repositories	$\square_1$	$\square_2$	$\square_3$	$\square_4$	$\square_5$

REVIEW OF DATA SHARING PLANS IN GRANT PROPOSALS

5. In the last 3 years, have you served as a reviewer of grant proposals for federal agencies that dealt with life science research grants (e.g., NIH, NSF, Department of Defense, Department of Energy)?

☐1 Yes
☐2 No → *Go to question <6>.*

6. How influential was the quality of the data sharing plans in your overall evaluation of the proposals you reviewed?

☐1 Very important
☐2 Important
☐3 Somewhat important
☐4 Not at all important

COMPLIANCE WITH MATERIAL TRANSFER AGREEMENTS

7. In the last 3 years, how often have you shared data or materials with someone outside your institution *without* a material transfer agreement (MTA), even though you knew such an agreement was required by your institution?

☐1 Never
☐2 Rarely
☐3 Sometimes
☐4 Always
☐5 Not applicable
☐6 I am not aware of policies at my institution requiring an MTA.

8. In those instances when you did not get an MTA from your institution, please indicate the importance of the following factors (asked only of those who answered 2, 3, or 4 to question <7>)

	Very important	Moderately important	Not very important	Not at all important
a. Scope of MTA overly broad	☐1	☐2	☐3	☐4
b. MTA takes too much time to arrange	☐1	☐2	☐3	☐4
c. MTA requires too much red tape at own institution	☐1	☐2	☐3	☐4
d. MTA negotiations between own and other institution too onerous	☐1	☐2	☐3	☐4
e. Philosophically opposed to MTA restrictions	☐1	☐2	☐3	☐4
f. Other (specify one) ____________	☐1	☐2	☐3	☐4

DATA SHARING IN GENETICS

9. In the last 3 years, has your lab conducted or collaborated on a project that generated primary data for a genome wide association study (GWAS)?

☐$_1$ Yes
☐$_2$ No → *Go to question <10>.*

10. In your most recently completed GWAS, were you required to deposit data generated from the study into a data repository (e.g., dbGaP)?

☐$_1$ Yes
☐$_2$ No → *Go to question <11>.*

11. In your most recently completed GWAS, did you deposit the required data into the data repository?

☐$_1$ Yes
☐$_2$ No → *Go to question <12>.*

12. Please indicate the amount of time that elapsed between the date that data from your most recently completed GWAS was submitted to the repository and the date you submitted your first publication using that data.

☐$_1$ Less than 6 months
☐$_2$ Between 6 and 12 months
☐$_3$ Between 12 and 24 months
☐$_4$ More than 24 months
☐$_5$ Data from the GWAS have not been included in any manuscripts submitted for publication

SANCTIONS FOR DATA WITHHOLDING

13. As a result of another academic scientist's failure to share information, data or materials, have you *ever…?*

	Yes	No
a. Appealed to a funding agency, journal, or professional association	☐$_1$	☐$_2$
b. Stopped collaborating with another academic scientist	☐$_1$	☐$_2$
c. Refused to share your information, data or materials with that person or group	☐$_1$	☐$_2$
d. Delayed sharing with that person or group	☐$_1$	☐$_2$
